# Association of Serum Biomarkers and Cardiac Inflammation in Patients With Atrial Fibrillation: Identification by Positron Emission Tomography

**DOI:** 10.3389/fcvm.2021.735082

**Published:** 2021-10-12

**Authors:** Bi-Xi Chen, Boqia Xie, Yang Zhou, Liang Shi, Yanjiang Wang, Lijun Zeng, Xingpeng Liu, Min-Fu Yang

**Affiliations:** ^1^Department of Nuclear Medicine, Beijing Chaoyang Hospital, Capital Medical University, Beijing, China; ^2^Cardiac Center, Beijing Chaoyang Hospital, Capital Medical University, Beijing, China

**Keywords:** atrial fibrillation, biomarker, epicardial adipose tissue, inflammation, fibrosis, ^18^F-fluorodeoxyglucose

## Abstract

**Background:** Peripheral biomarkers may be affected by various factors, their reliability in reflecting local cardiac inflammatory status in patients with atrial fibrillation (AF) needs further exploration. This prospective study was aimed to investigate the relationship between circulating biomarkers and local cardiac inflammation measured by epicardial adipose tissue (EAT) activity *via* ^18^F-fluorodeoxyglucose (FDG) imaging in AF patients.

**Methods:** From 2017 to 2018, 83 AF patients [43 persistent AF (PsAF) and 40 paroxysmal AF (PAF)] referred for radiofrequency catheter ablation (RFCA) were recruited. Pre- and post-RFCA blood samples were collected to measure IL-6, IL-8, IL-10, IL-18, TNF-α, Hsp27, Hsp60, Hsp70, PDGF-BB, MMP-2, MMP-9, MPO, TGF-β1, Gal-3, and sST2. Pre-RFCA FDG images were obtained to assess EAT activity. Sixty-seven patients (35 PAF and 32 PsAF) received RFCA were regularly followed for 27 (24, 29) months.

**Results:** Higher hsCRP and IL-6 and lower TGF-β1 were demonstrated in PsAF patients compared with PAF patients. Multivariate logistic regression analysis demonstrated that Gal-3 (OR: 1.221, 95% CI: 1.024–1.456, *P* = 0.026) and MPO (OR: 1.002, 95% CI: 1.001–1.003, *P* = 0.027) were independently correlated with EAT activity. The percentage decrease of Hsp60 linearly correlated with that of EAT activity post-RFCA (Spearman *r*_s_ = 0.455, *P* = 0.019). Seventeen patients (10 PsAF and 7 PAF) had AF recurrence, but none of the selected biomarkers were predictive of post-RFCA recurrence.

**Conclusion:** Our findings demonstrated that in patients with AF, Gal-3 correlated with local cardiac inflammation, and Hsp60 was associated with the alleviation of cardiac inflammation after RFCA.

## Introduction

Atrial fibrillation (AF) is the most common sustained arrhythmia increasing with age, and it is an independent risk factor for stroke and death. Recently, biomarkers with the potential value in assisting treatment decision-making and prognostic stratification of AF have been addressed in multiple studies. Of these, inflammatory biomarkers are popular indices because they have been suggested to correlate with atrial electrical and structural remodeling (1). However, due to the lack of information on these indicators in the management of AF, to date, biomarkers are only given a class IIb recommendation with a level of evidence B for further refined stroke and bleeding risk in the AF guidelines (2). Biomarkers of AF need to be further investigated to better understand their potential utility in AF management.

Inflammation is an essential factor in the occurrence and persistence of AF (3), but it is unclear whether peripheral biomarkers are reliable to reflect local cardiac inflammatory status. Plasma biomarkers are prone to be affected by various factors, and previous studies showed a significant difference between intracardiac and peripheral samples (1). There are scarce feasible techniques for assessing cardiac inflammation, apart from invasive biopsy. Recently, ^18^F-fluorodeoxyglucose (FDG) positron emission tomography (PET) imaging has gained increased attention for its ability to detect various inflammatory diseases (4). Its value has also been explored in AF patients demonstrating that elevated FDG activity in the epicardial adipose tissue (EAT), local cardiac source of inflammatory cytokines, could be regarded as a marker of local cardiac inflammation (5, 6). Therefore, in this prospective study, we adopted cardiac FDG PET imaging to investigate the correlation between peripheral blood indicators and local cardiac inflammation in AF patients. After reviewing previous studies of AF biomarkers, 15 biomarkers were selected (IL-6, IL-8, IL-10, IL-18, TNF-α, Hsp27, Hsp60, Hsp70, PDGF-BB, MMP-2, MMP-9, MPO, TGF-β1, Gal-3, and sST2).

## Materials and Methods

### Study Population

The study population derived from a prospective AF study (7) that was approved by the Institutional Ethics Committee of Beijing Chaoyang Hospital. The enrollment period was from August 2017 to August 2018. A total of 83 consecutive AF patients referred to the arrhythmic ward of Beijing Chaoyang Hospital were prospectively enrolled.

Considering the exploratory nature of the present study, which aimed to investigate the relationship between circulating biomarkers and local cardiac inflammation assessed by FDG, AF patients with significant comorbidities were excluded to minimize potential fluctuations of biomarkers and myocardial FDG uptake caused by other pathologies. The exclusion criteria included unstable ischemic heart disease, acute coronary artery disease, prior myocardial infarction, severe valvular heart disease, cardiomyopathy, congenital heart disease, decompensated heart failure, prior cardiac surgery, history of radiofrequency ablation therapy, pulmonary arterial hypertension, hyperthyroidism, end-stage renal disease, infectious disease, inflammatory disease, malignant disease, or unstable clinical status.

Twenty age- and gender-matched subjects were selected from our whole-body FDG PET/CT database (following diet preparation of > 16 h fast) to establish the normal range of FDG uptake in EAT. The inclusion criteria for the control group were as follows: (1) no history of AF; (2) no history of malignancy; and (3) no malignant or inflammatory findings on PET/CT imaging. Informed consent was signed by all eligible AF patients before enrollment.

### Study Procedures

All patients underwent fasting baseline FDG imaging within 2 days before RFCA based on individualized evaluation. Venous blood samples were collected from the cubital vein in the morning of FDG imaging. After comprehensive clinical evaluation, 70 patients [36 paroxysmal AF (PAF) and 34 persistent AF (PsAF)] received RFCA. Pre-RFCA transthoracic echocardiography was routinely performed in all patients. The left atrium volume index (LAVI) and right atrium (RA) area were obtained from transthoracic echocardiographic measurement (8).

### PET/CT Imaging and Interpretation

A combined strategy was employed to suppress physiological myocardial FDG uptake as described previously. In brief, after a prolonged fasting (>16 h), unfractionated heparin (50 IU/kg) was administered intravenously 15 min before FDG injection (anticoagulant medication was withdrawn in the morning). A 16-slice PET/CT scanner (Discovery STE, GE, USA) was used to acquire FDG PET/CT images 60 min after intravenous injection of 3.7 MBq/kg of FDG. The CT parameters were as follows: 140 kV, 120 mA, pitch 1.375, 16 × 0.625 mm collimation, and section width 5 mm. Two beds of PET images (5 min/bed, 3D mode) were acquired, and the heart was centered in the view. In control subjects, imaging was performed (2-3 min/bed, 3D mode) 60 min after intravenous injection of 3.7 MBq/kg of FDG, all patients underwent CT (140 kV, 120 mA) for attenuation correction. Attenuation-corrected PET images (voxel size, 3.9 × 3.9 × 3.3 mm) were reconstructed from the CT data using a 3D ordered-subset expectation maximization algorithm (14 subsets, 2 iterations). Integrated PET and CT images were obtained automatically on AW VolumeShare 2 (GE Healthcare). PET/CT images were independently interpreted by two nuclear physicians blinded to clinical data. EAT adjacent to the origin of the right coronary artery was selected to measure FDG activity (6), and the maximum standardized uptake value (SUV_max_) was recorded. The upper normal limit of EAT SUV_max_ was calculated as the mean plus 2 standard deviations (SD) from the control group. According to the pre-defined study protocol, patients presenting with abnormal atrial FDG uptake (FDG uptake in the atrial structure higher than in the blood pool) before RFCA were scheduled for repeated PET/CT imaging at 3 months post-ablation. FDG activity in the atrium and EAT were evaluated at baseline and follow-up scans.

The measurement protocol of FDG activity in the atrium was described in our previous study (7). In brief, visual insepction was adopted first: FDG uptake in the atrial structure that was higher than in the blood pool was determined as abnormal. Second, quantitatively analyzed was used to evaluate the FDG uptake values in the atrial structures and EAT. Visible uptake of the atrial wall on each PET transaxial image was measured by placing region of interest (ROI) guided by CT. The maximum standardized uptake value (SUVmax) out of all slices was selected to represent the activity of the atrium. In case no visible uptake could be analyzed, a circular ROI (5-mm in diameter) was placed on the right lateral wall of the LA at the level of the right inferior pulmonary vein and on the right lateral wall of the RA at the level of the aortic root. A ROI around the AA on transaxial sections was used to measure the activity of the atrial appendage (AA). ROI placed on the LA and RA cavity and the mean SUV (SUVmean) were used to calculate the background value of FDG uptake and the value of target-to-background ratio (TBR). ROI was placed on the region adjacent to the origin of right coronary artery to assess the EAT activity.

### Radiofrequency Catheter Ablation

An experienced electrophysiologist (XPL), blinded to serum biomarkers and PET/CT imaging results, performed all catheter ablation procedures. Deflectable multipolar catheter (PentaRay® Nav: 20 poles, 2-6-2 mm electrode spacing, Biosense Webster, Inc.) was applied for voltage mapping with 3D electroanatomic mapping system CARTO (Biosense Webster, Diamond Bar, USA). Pulmonary vein isolation (PVI) was first performed for all AF patients using a 3.5 mm irrigated-tip quadripolar ablation catheter (Smart Touch, 2-5-2 mm inter-electrode spacing, D/F or F/J curves, Biosense Webster, Inc.), and additional ablation was continued if AF did not terminate after PVI. End-point of ablation was termination of AF and sinus rhythm restoration.

### Biomarker Analysis

Biomarkers were analyzed in AF patients. Serum was separated from venous blood sample by centrifugation at 4°C for 10 min at 4,000 rpm within 2 h of collection. The serum sample was stored at −80°C until analysis. Biomarkers were analyzed using commercially available ELISA kits (R&D Systems Inc., Minneapolis, MN) in accordance with the manufacturer's recommendations. Besides the selected biomarkers, highly sensitive C-reactive protein (hsCRP), B-type natriuretic peptide (BNP), and low-density lipoprotein (LDL) were routinely measured in AF patients. The above biomarkers were reassessed in the morning of the follow-up PET imaging 3 months after RFCA in 26 AF patients.

### Follow-Up

After RFCA, all patients received oral anticoagulants (warfarin or new oral anticoagulants) for a minimum of 3 months. Post-RFCA antiarrhythmic drugs were administered based on individual conditions. Antiarrhythmic medications were discontinued at 12 weeks post-ablation if no recurrence was recorded.

Before discharge, all patients were instructed to contact follow-up staff if they developed symptoms. The social-networking software, WeChat, was applied to connect and collect information provided by patients during the follow-up. Moreover, 24-h Holter monitoring was scheduled at 1, 3, 6, 12, 24, and 36 months following the ablation. Early recurrence (blanking period recurrence) was defined as recurrence during the first 3 months, whereas late recurrence was defined as recurrence after the third month post-ablation.

### Statistical Analysis

SPSS Statistics (Version 24; IBM) was used to perform the statistical analyses. The normality of distribution was assessed using the Kolmogorov–Smirnoff-test. Continuous variables were described as mean ± SD or medians (interquartile ranges) depending on the normality of distribution. Categorical variables were expressed as absolute numbers and percentages. Variables between groups were compared using Student's *t*-test, Mann-Whitney *U*-test, Chi-square test, or Fisher exact-test, depending on the nature of the data. Spearman's correlation analysis was conducted to determine the correlation between the percentage change of biomarkers and that of the EAT activity before and post-RFCA. Multivariate logistic regression models were used to calculate the relevant biomarkers of enhanced EAT activity among biomarkers with a *P*-value < 0.05 in the bivariate analysis. Cox regression analysis was used to determine the predictors of AF recurrence. Collinearity diagnostic test was adopted to examine the presence of significant interactions and to identify possible multicollinearity of the covariates. Intra- and interobserver reproducibility of EAT FDG activity measurement was assessed using the intraclass correlation coefficient (ICC), which demonstrated excellent reproducibility (all ICC > 0.8) (Supplementary Table 1). *P*-values < 0.05 were considered statistically significant.

## Results

### Patients' Characteristics

Clinical data of the AF patients are summarized in Table 1. The median age was 69 (60, 75) years in PsAF group (67% men) and 62 (55, 78) years in PAF (55% men) group. PsAF patients had higher BMI, more frequent hypertension and diabetes, larger left and right atrium, and lower LVEF compared with PAFs (*P* < 0.05). In control subjects, the median age was 67 (63, 68) years, the mean BMI was 25.4 ± 3.5, 60% was men. Four subjects (20%) with coronary artery disease, 5 (25%) with diabetes mellitus, 10 (50%) with hypertension, 2 (10%) with peripheral vascular disease, and 1 (5%) with stroke. The above characteristics were not significantly different from AF patients.

**Table 1:** Patients' characteristics.

	**AF (*n* = 83)**	**PsAF (*n* = 43)**	**PAF (*n* = 40)**
Age, years	67 (56, 74)	69 (60, 75)	62 (55, 78)
Male (%)	51 (61)	29 (67)	22 (55)
BMI, kg/m^2^	26.2 ± 3.1	27.1 ± 3.4*	25.2 ± 2.5
Hypertension (%)	52 (63)	32 (74)*	20 (50)
Coronary artery disease (%)	24 (29)	9 (21)	15 (38)
Peripheral vascular disease (%)	22 (27)	9 (21)	13 (33)
Diabetes mellitus (%)	31 (37)	23 (53)*	8 (20)
Stroke (%)	20 (24)	13 (30)	7 (18)
CHA2DS2-VASc score	3 (2, 5)	3 (1,4)	0.136
Echocardiography			
LAVI, ml/m^2^	27.8 (20.3, 33.2)	31.8 (27.8, 34.4)*	20.0 (15.9, 27.8)
RA area, cm^2^	19.7 ± 4.6	22.5 ± 3.5*	16.6 ± 3.5
LVEF (%)	66 ± 7	63 ± 6*	68 ± 6
LA activity	1.1 (1.0, 1.2)	1.1 (1.0, 1.3)	1.0 (1.0, 1.1)
LAA activity	1.0 (0.9, 1.1)	1.1 (0.9, 1.2)*	0.9 (0.8, 1.0)
RA activity	1.1 (1.0, 1.4)	1.4 (1.1, 1.7)*	1.1 (0.9, 1.1)
SUV_max_-EAT	1.4 (1.2, 1.6)	1.5 (1.3, 1.6)	1.3 (1.1, 1.5)
Serum biomarkers			
hsCRP, pg/mL	1.3 (0.7, 2.6)	1.5 (0.8, 3.1)*	1.0 (0.6, 2.0)
BNP, mg/L	124.0 (51.0, 269.0)	174.0 (124.0, 290.0)*	54.0 (24.3, 143.0)
LDL, mmol/L	2.3 ± 0.8	2.3 ± 0.8	2.4 ± 0.8
IL6, pg/ml	5.9 (4.1, 8.2)	6.3 (4.3, 9.3) *	5.3 (3.3, 6.5)
IL8, pg/ml	15.2 (7.8, 24.8)	15.2 (6.2, 24.1)	15.7 (9.7, 25.1)
IL10, pg/ml	12.1 ± 7.2	11.2 ± 7.6	13.0 ± 6.7
IL18, pg/ml	51.9 (35.3, 84.6)	42.8 (33.4, 78.5)	57.6 (36.9, 87.6)
TNF-α, pg/ml	5.7 (2.9, 12.2)	5.9 (3.2, 13.3)	5.2 (2.4, 10.4)
Hsp27, ng/ml	139.8 ± 73.0	147.2 ± 73.4	129.8 ± 72.2
Hsp60, ng/ml	52.4 ± 19.7	52.9 ± 30.2	51.7 ± 19.3
Hsp70, ng/ml	23.5 ± 11.9	23.5 ± 12.0	23.6 ± 11.9
TGF-β1, ng/ml	8.9 ± 2.7	8.2 ± 2.5*	9.6 ± 2.5
PDGF-BB, ng/ml	3.3 (2.7, 4.6)	3.4 (3.0, 4.6)	3.3 (2.4, 5.1)
sST2, ng/ml	12.1 (8.1, 16.2)	13.9 (7.9, 18.2)	11.6 (8.1, 15.1)
Gal-3, ng/ml	7.0 (5.6, 9.6)	7.4 (5.6, 9.6)	6.1 (5.3, 8.3)
MMP-2, ng/ml	245.8 ± 103.7	259.7 ± 102.5	230.8 ± 104.1
MMP-9, ng/ml	1139.9 ± 602.2	1134.9 ± 650.1	1145.5 ± 554.4
MPO, ng/ml	1178.1 ± 360.2	1188.5 ± 345.4	1166.9 ± 379.7

P < 0.05 (*PsAF vs. PAF).

*BMI, body mass index; LAVI, left atrium volume index; RA, right atrium; SUV_max_, maximum standardized uptake value; EAT, epicardial adipose tissue*.

### Biomarkers in PsAF and PAF

The results of the serum biomarkers are shown in Table 1. Individuals with PsAF had higher hsCRP (*P* = 0.02), IL6 (*P* = 0.02), and lower TGF-β1 levels (*P* = 0.018) than PAF patients.

### Correlation Between Biomarkers and EAT Activity

**Figure 1:**
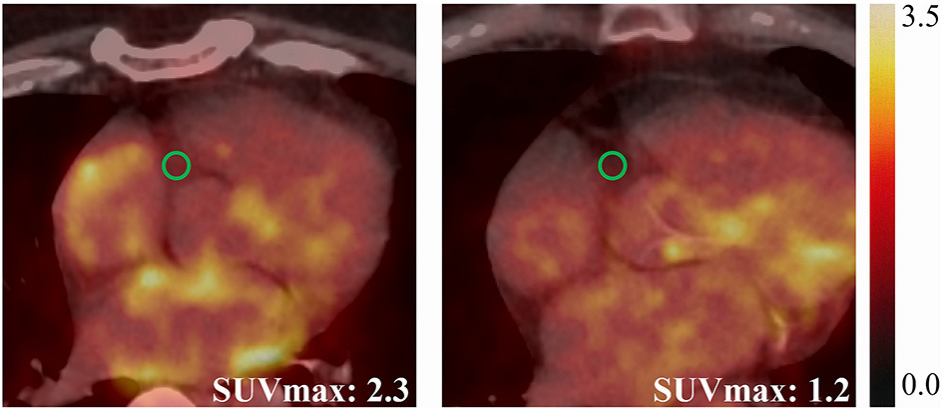
Representative cases showing elevated EAT activity (SUV_max_ = 2.3) in a 57-year-old male PsAF patient (left), and normal EAT activity (SUV_max_ = 1.2) in an 80-year-old male PAF patient (right).

The mean SUV_max_ of EAT in control subjects was 1.18 ± 0.14. Based on the pre-defined definition, the cut-off value of SUV_max_ for determining increased EAT uptake was 1.46. As a result, 30 AF patients (36.1%) had abnormally enhanced EAT activity. The characteristics of the AF participants with and without increased EAT activity (Figure 1) are summarized in Table 2. Univariate analysis demonstrated that patients with increased EAT activity had higher levels of BNP, Gal-3, and MPO (all *P* < 0.05). After multivariate logistic regression analyses (adjusted for PsAF type, BNP, LAVI, and RA area), Gal-3 and MPO remained independently correlated with enhanced EAT activity [Gal-3 (odds ration OR: 1.221, 95% CI: 1.024–1.456, *P* = 0.026); MPO (OR: 1.002, 95% CI: 1.001–1.003, *P* = 0.027)]. Subgroup analysis according to gender, age, LA size, and RA size were presented in Supplementary Tables 2–5. In addition, in line with our previous study (7), significant correlation was found between EAT activity and LA (*r*_s_ = 0.504, *P* < 0.001), LAA (*r*_s_ = 0.467, *P* < 0.001), and RA (*r*_s_ = 0.561, *P* < 0.001).

**Table 2:** Comparison between patients with- and without increased EAT activity.

**Variables**	**Increased EAT activity (*n* = 30)**	**Normal EAT activity (*n* = 53)**	***P-*value**
Age, year	66 ± 9	64 ± 12	0.315
Male (%)	19 (63)	32 (60)	0.790
PsAF (%)	21 (70)	22 (42)	**0.013**
BMI, kg/m^2^	25.9 ± 1.9	26.4 ± 3.6	0.463
Blood glucose, mmol/L	113.4 (100.8, 142.2)	111.6 (102.6, 127.8)	0.547
Hypertension (%)	15 (50)	37 (70)	0.073
Coronary artery disease (%)	9 (30)	15 (28)	0.870
Peripheral vascular disease (%)	7 (23)	15 (28)	0.622
Diabetes mellitus (%)	11 (37)	20 (38)	0.549
Stroke (%)	8 (27)	12 (23)	0.680
LA activity	1.2 (1.0, 1.8)	1.1 (1.0, 1.2)	**0.022**
LAA activity	1.1 (1.0, 1.6)	1.0 (0.9, 1.1)	** <0.001**
RA activity	1.6 (1.1, 1.9)	1.1 (1.0, 1.4)	** <0.001**
Echocardiography			
LAVI, ml/m^2^	30.9 (27.5, 34.5)	26.2 (18.3, 32.7)	**0.006**
RA area, cm^2^	21.2 ± 5.0	18.8 ± 4.1	**0.025**
LVEF (%)	64.3 ± 6.4	66.4 ± 6.6	0.179
Serum biomarkers			
hsCRP, pg/mL	1.5 (0.8, 2.8)	1.1 (0.6, 2.3)	0.209
BNP, mg/L	212.0 (83.7, 311.5)	117.0 (33.0, 196.0)	**0.033**
LDL, mmol/L	2.3 ± 0.9	2.3 ± 0.8	0.967
IL-6, pg/ml	6.3 (4.1, 9.1)	5.6 (4.0, 7.7)	0.471
IL-8, pg/ml	15.2 (6.7, 24.2)	16.3 (8.2, 25.2)	0.599
IL-10, pg/ml	11.7 ± 7.9	12.1 ± 7.3	0.900
IL-18, pg/ml	51.3 (35.2, 79.6)	52.7 (36.9, 85.6)	0.924
TNF-α, pg/ml	7.7 (3.3, 13.6)	5.2 (2.3, 11.9)	0.388
Hsp27, ng/ml	138.8 ± 38.4	140.4 ± 73.5	0.927
Hsp60, ng/ml	54.1 ± 20.5	51.4 ± 19.3	0.551
Hsp70, ng/ml	24.3 ± 11.3	23.1 ± 13.4	0.636
TGF-β1, ng/ml	8.5 (6.9, 10.9)	8.8 (6.3, 10.5)	0.583
PDGF-BB, ng/ml	3.2 (2.6, 4.4)	3.4 (2.6, 5.2)	0.495
sST2, ng/ml	12.9 (10.0, 16.6)	12.1 (7.6, 16.9)	0.391
Gal-3, ng/ml	7.9 (5.9, 10.7)	6.0 (5.0, 8.9)	**0.023**
MMP-2, ng/ml	257.6 ± 90.7	239.1 ± 110.6	0.439
MMP-9, ng/ml	1263.9 ± 587.5	1075.5 ± 606.3	0.197
MPO, ng/ml	1305.9 ± 386.9	1105.8 ± 326.2	**0.014**
Early recurrence^*^	7 (32)	7 (16)	0.223
Late recurrence^*^	8 (36)	9 (20)	0.148

*BMI, body mass index; LAVI, left atrium volume index; RA, right atrium; SUV_max_, maximum standardized uptake value; EAT, epicardial adipose tissue*.

* *Sixty-seven patients underwent follow-up, 22 with increased EAT activity and 45 without. The bold values are < 0.05*.

### Correlation Between Biomarkers and RFCA

AF termination was achieved in 25 of 34 (74%) PsAF patients, while 9 patients converted to atrial flutter (26%) and were successfully terminated by additional ablation. Comparisons of biomarkers in PsAF patients with and without successful AF termination are listed in Supplementary Table 6, but none of them showed statistically significant differences.

**Figure 2:**
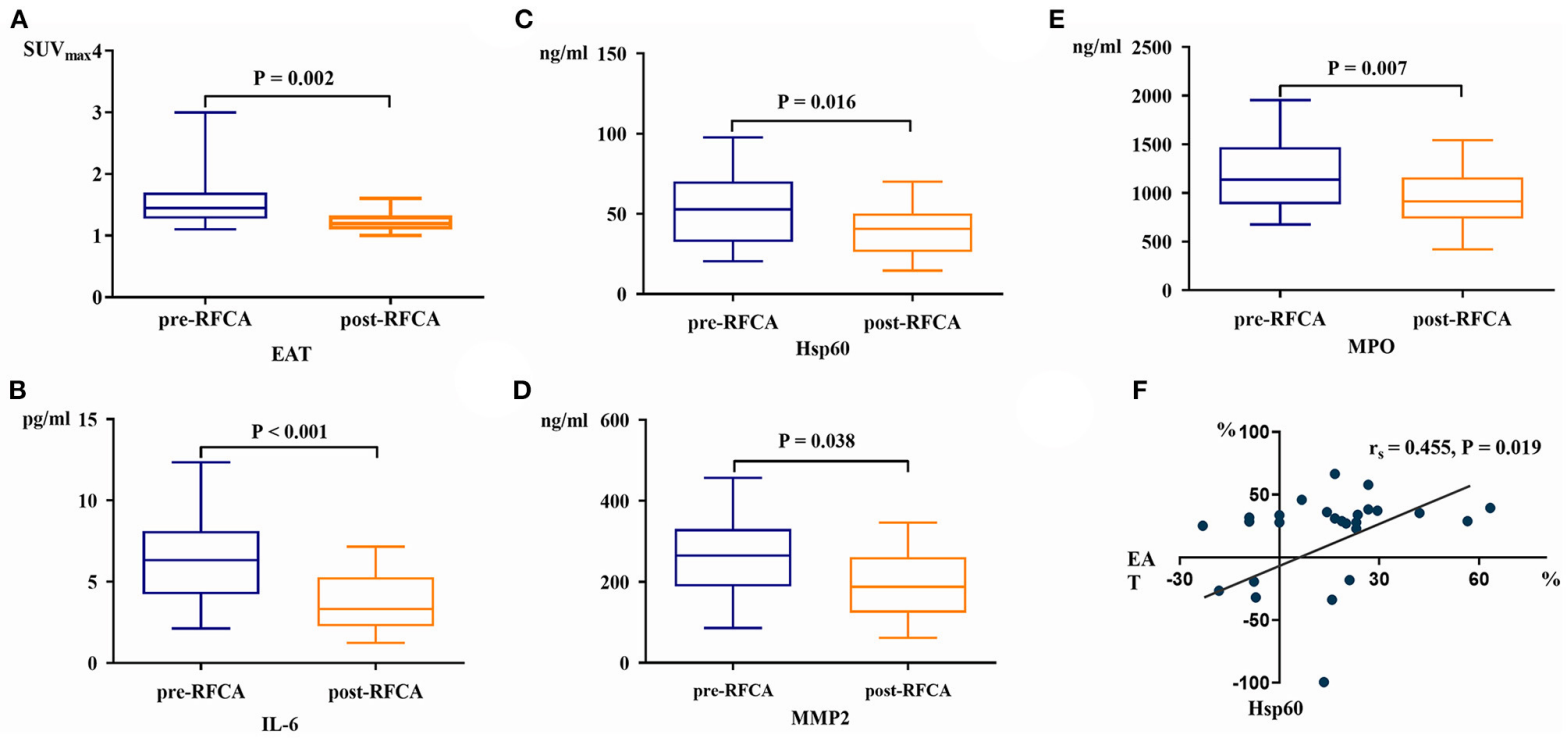
The EAT activity **(A)**, serum IL-6 **(B)**, Hsp60 **(C)**, MMP2 **(D)**, and MPO levels **(E)** all decreased significantly after RFCA. The percentage change of Hsp60 was linearly correlated with that of EAT activity post-RFCA **(F)**.

Twenty-six patients (19 PsAF and 7 PAF) underwent repeated serum biomarkers analysis and FDG imaging 3 months after ablation. The EAT activity decreased significantly after RFCA [SUV_max_-EAT: 1.5 (1.3, 1.7) vs. 1.2 (1.1, 1.3), *P* = 0.002] (Figure 2A), and so did the levels of IL-6, Hsp60, MMP2, and MPO (all *P* < 0.05) (Figures 2B–E). Spearman correlation analysis demonstrated that only the percentage decrease of Hsp60 significantly correlated with that of EAT activity (*r*_s_ = 0.455, *P* = 0.019) (Figure 2F).

### Follow-Up

**Figure 3:**
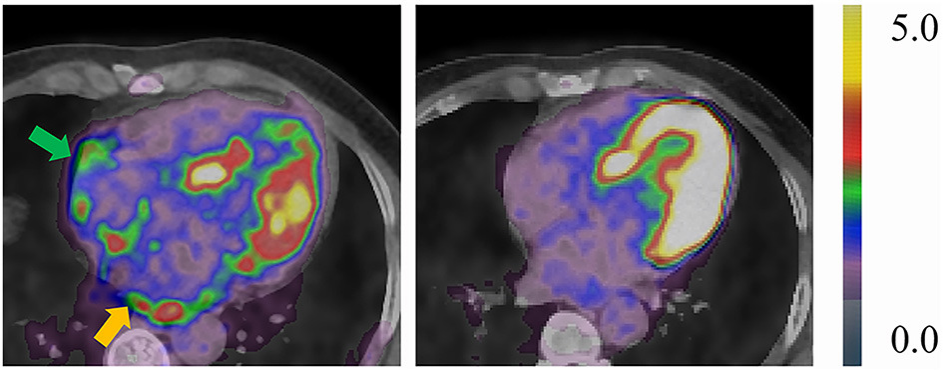
Representative case of a 49-year-old male PsAF patient before (left: TBR_LA_ = 2.16, TBR_RA_ = 1.79) and after RFCA (right: TBR_LA_ = 0.69, TBR_RA_ = 1.36). Yellow arrow indicates abnormal LA FDG uptake, green arrow indicates abnormal RA FDG uptake. Both LA and RA FDG activities reduced compared to baseline scans.

Of the 70 patients receiving RFCA, 3 patients (1 PAF and 2 PsAF) were lost to follow-up, while the remaining 67 patients were followed for 27 (24, 29) months. The most commonly prescribed antiarrhythmic medication was amiodarone (58/70), followed by propafenone (4/70), and a combination of those two (1/70). Fourteen patients (11 PsAF and 3 PAF) experienced early recurrence, and they all received synchronized cardioversion; in contrast, 17 patients (10 PsAF and 7 PAF) had a late recurrence. None of the biomarkers were predictive of early or late recurrence (Supplementary Tables 7, 8), even though borderline significance was identified in sST2 (HR: 0.916, 95% CI: 0.839–1.001, *P* = 0.053). Significant reduction of FDG activity was observed in LA, RA, and EAT (all *P* < 0.05) (Table 3; Figure 3). Correlation analysis of post-RFCA FDG uptake (in EAT, LA, LAA, and RA) and the difference between pre- and post-RFCA (pre-uptake – post-uptake) with early and late recurrence were performed and identified higher post-RFCA LA activity in patients without early recurrence (*P* = 0.012) (Supplementary Tables 9, 10).

**Table 3:** Comparisons of FDG activities pre- and post-RFCA in the atrium and EAT.

	**Pre-RFCA**	**Post-RFCA**	***P-*value**
**TBR**			
LA	1.2 (1.0, 1.6)	0.8 (0.7, 0.9)	0.001
LAA	1.1 (1.0, 1.4)	1.0 (0.9, 1.2)	0.051
RA	1.4 (1.3, 1.7)	1.1 (0.9, 1.2)	0.001
**SUV**_**max**_			
EAT	1.5 (1.3, 1.7)	1.2 (1.1, 1.3)	0.002

*TBR, Target-to-background tissue; SUV_max_, maximum standardized uptake value; EAT, epicardial adipose tissue*.

## Discussions

In this prospective study, we explored the clinical significance of the reported serum biomarkers in patients with AF. Although blood sample obtained in the left atrium would be better than the peripheral veins, the latter was less invasive and most commonly used. The main findings include the following: (1) among the targeted biomarkers, hsCRP and IL-6 were higher and TGF-β1 was lower in PsAF patients than in PAF patients; (2) Gal-3 correlated with local cardiac inflammation, while Hsp60 was associated with the alleviation of cardiac inflammation after RFCA; (3) none of the studied biomarkers could predict successful sinus rhythm maintenance by RFCA and post-RFCA recurrence.

### Biomarkers and Local Cardiac Inflammation

EAT is a metabolically active organ producing inflammatory cytokines, which can diffuse into the adjacent atrial tissue and contribute to the genesis of AF. As reported in the Framingham Heart study (9), EAT volume quantified by CT was an independent predictor of AF. Similarly, by using FDG PET imaging, both Mazurek et al. (5) and our group (6) showed an enhanced EAT activity in patients with AF. These findings consistently suggest that EAT is a local source of inflammation during the pathological processes of AF. Gal-3 is a fibrotic biomarker mainly produced by macrophages and other cell types including adipocytes, and it is upregulated in growing adipose tissue and during inflammation. Ionin et al. (10) reported strong correlation between epicardial fat thickness and Gal-3 in AF patients (*r* = 0.612; *P* < 0.001). In contrast, myeloperoxidase (MPO)—a hemoprotein produced by polymorphonuclear neutrophils and macrophages—is a crucial regulator of activation of pro-MMPs and consequent atrial collagen deposition, which results in AF. Karatas A et al. found a correlation of serum levels of MPO and EAT thickness in patients with chronic kidney disease (11). Considering EAT activity might be affected by age, gender, heart failure, and atrial size, we performed subgroup analysis to adjust for gender, age, AF type, BNP, LA, and RA size, and identified that Gal-3 and MPO remain independently correlated with enhanced EAT activity. Accordingly, we presume that Gal-3 and MPO are related to EAT. However, given low OR of MPO, MPO cannot be regarded as a reliable marker. Therefore, our data suggested Gal-3 may be used as surrogates for local cardiac inflammatory status.

### Biomarkers Related to AF Duration

The relationship between serum levels of biomarkers and the duration of AF remains controversial. The studies comparing PsAF and PAF patients have reported that systemic biomarkers including hsCRP, IL-6, IL-8, IL-10, IL-18, TNF-α, TGF-β1, PDGF-BB, sST2, Gal-3, and MMP-9 were all elevated in PsAF patients, while Hsp27, Hsp70, and MMP-2 were higher in PAF patients (12, 13). However, in the present study, only IL-6 and hsCRP were higher in PsAF compared with PAF patients. The discrepancy may be partially explained by the exclusion criteria of our study, which minimized the influence of pathologies other than AF.

IL-6 is an upstream inflammatory cytokine leading to the hepatic synthesis of downstream CRP; hence, elevated IL-6 and hsCRP in PsAF patients suggest a more prominent inflammatory reaction in prolonged AF exposure. Therefore, IL-6 and hsCRP may be used as reference biomarkers to assess the duration of atrial fibrillation.

In contrast to some reports, we found that TGF-β1 level was lower in PsAF than in PAF patients. Being a key player in tissue fibrosis, TGF-β1 promotes and controls the production of extracellular matrix, which results in atrial fibrosis and contributes to the development of AF. Our finding regarding the TGF-β1 level may be due to its biphasic response during atrial fibrogenesis, which shows an early increase in PAF and a later decrease in PsAF patients (14). In other words, the downregulated TGF-β1 in PsAF patients may result from a higher consumption within the remodeled and fibrotic atrium. However, as these are merely speculations, further studies are needed to clarify the underlying mechanism.

### Changes in Biomarkers After RFCA

The inflammatory indices of IL-6 and MPO, the fibrotic markers of PDGF-BB and MMP2, and the stress protein Hsp60 dropped significantly when AF was terminated by RFCA. These findings indicate that the above biomarkers have an “on-and-off” intercorrelation in the presence of AF. Moreover, among these indices, the change in Hsp60 significantly correlated with the change in EAT activity pre- and post-RFCA. It has been demonstrated that Hsp60 is highly expressed in cardiac tissues and that it can be released into circulation under stress, thereby acting as an inflammatory danger signal and inducing secretion of proinflammatory mediators from adipocytes. We suppose that AF termination reduces Hsp60 expression and alleviates the downstream EAT activity. However, the specific intercorrelation needs further research.

In the present study, significant reduced FDG activity was observed both in the atrium and EAT compared with baseline scans. This suggests that both atrial and EAT FDG uptake are reversible once AF is terminated. Although conclusive causal relationship between AF and FDG uptake cannot be established due to the small sample size and the lack of tissue-leveled evidence, we assume sinus rhythm should always be the therapeutic goal.

### Biomarkers and Their Clinical Significance for RFCA

None of the tested biomarkers were found related to post-RFCA recurrence. The only borderline significance was recognized for sST2 (*P* = 0.053), which exhibited a protective effect on post-RFCA prognosis. sST2 is a relatively new biomarker, which is released from the myocardium in response to pressure or volume overload. The implications of sST2 are mainly explored in the fields of acute coronary syndrome and heart failure, and it has been suggested that sST2 is a biomarker with diagnostic and/or prognostic value in coronary artery disease, myocardial infarction, heart failure, and ischemic stroke. However, the effect of sST2 on AF pathogenesis has not been widely investigated. In a study by Rienstra et al. (15), no significant association was found between sST2 and new onset of AF. In contrast, Okar et al. (16) reported that elevated levels of sST2 were related to AF recurrence after cryoballoon catheter ablation in PAF patients. Our finding is different from the aforementioned studies given that lower sST2 was associated with higher recurrence rate in PsAF patients. Recent evidence supports that sST2 has direct anti-inflammatory actions on macrophages through downregulating Toll-like receptors (17). Hence, we suppose that the anti-inflammatory action exerted by sST2 may contribute to the low recurrence of AF, but further research is needed to illustrate the prognostic value of sST2 in PsAF patients.

According to our findings, although the biomarkers are essential in the biological process of inflammation and fibrosis, they were unrelated to the recurrence of AF after RFCA. Therefore, these biomarkers should be cautiously used for the prediction of prognosis.

### Atrial FDG Uptake in PsAF

In the present study, atrial FDG uptake was found related to EAT activity, which was in line with our previous researches exploring the clinical significance of atrial FDG accumulation in AF patients (6, 7). In short, we demonstrated atrial FDG accumulation was common in PsAF patients, and LA activity was related to that of EAT, and was inversely correlated to LA fibrosis. This evidence suggested local inflammation measured by EAT activity may play an essential role in the occurrence and persistence of atrial fibrillation, which may be assessed by peripheral Gal-3 level as been proved in the current study.

### Limitations

There were several limitations in this study that could be addressed in future research. First, the sample size was relatively small. Second, in order to minimize other pathological effects on biomarkers, patients with significant comorbidities were excluded; although these measures helped in securing the biomarkers specific to AF, they may have led to selection bias. Third, the study population was derived from a prospective AF study (7), according to the study protocol, only patients with abnormal atrial FDG uptake had a 3-month biomarkers reassessment, which may lead to selection bias. Finally, histological examination was not performed, and the underlying mechanism needs further investigation. Larger-scale studies with a more inclusive AF etiology and tissue-level evidence should be conducted to complement and extend the present conclusions.

In conclusion, in AF patients, circulating Gal-3 level correlates with local cardiac inflammation, and Hsp60 is associated with alleviation of cardiac inflammation after RFCA.

## Data Availability Statement

The raw data supporting the conclusions of this article will be made available by the authors, without undue reservation.

## Ethics Statement

The studies involving human participants were reviewed and approved by Institutional Ethics Committee of Beijing Chaoyang Hospital. The patients/participants provided their written informed consent to participate in this study.

## Author Contributions

BX and B-XC wrote the draft of the manuscript. BX, YZ, LS, YW, and LZ collected and analyzed the clinical data. B-XC analyzed the PET/CT data. BX analyzed the biomarker data. M-FY and XL conceived the study and interpreted the results. M-FY makes critical revision of the manuscript for important intellectual content. All authors contributed to the article's revision, agreed to its submission, and had full access to original data.

## Funding

This study was supported by the National Natural Science Foundation of China (81871380) and the Beijing Hospitals Authority Clinical Medicine Development of Special Funding Support (ZYLX202105).

## Conflict of Interest

The authors declare that the research was conducted in the absence of any commercial or financial relationships that could be construed as a potential conflict of interest.

## Publisher's Note

All claims expressed in this article are solely those of the authors and do not necessarily represent those of their affiliated organizations, or those of the publisher, the editors and the reviewers. Any product that may be evaluated in this article, or claim that may be made by its manufacturer, is not guaranteed or endorsed by the publisher.
