# Supplementary material for: Association of Serum Biomarkers and Cardiac Inflammation in Patients With Atrial Fibrillation: Identification by Positron Emission Tomography
Source: Front Cardiovasc Med. 2021 Oct 12;8:735082. doi: 10.3389/fcvm.2021.735082 (PMC8546267; doi:10.3389/fcvm.2021.735082)
Supplement: Supplementary file 1 [file Data_Sheet_1.docx]

**SUPPLEMENTAL MATERIALS**

Table of content

**Supplement Table S1.** Intra- and inter-observer reproducibility of EAT FDG activity measurement

**Supplementary Table S2.** Subgroup analysis of biomarkers according to LA size

**Supplementary Table S3.** Subgroup analysis of biomarkers according to RA size

**Supplementary Table S4.** Subgroup analysis of biomarkers according to age

**Supplementary Table S5.** Subgroup analysis of biomarkers according to gender

**Supplement Table S6.** Comparison between patients achieved AF termination by RFCA and those without

**Supplementary** **Table** **S7.** Cox regression analysis of predictor of AF recurrence

**Supplementary** **Table** **S8.** Comparison between AF with- and without early recurrence

**Supplemental Table S9.** Comparisons between patients with- and without early recurrence

**Supplemental Table S10.** Comparisons between patients with- and without late recurrence

**Supplement Table S1.** Intra- and inter-observer reproducibility of EAT FDG activity measurement

|  | Inter-observer ICC | Intra-observer ICC |
| --- | --- | --- |
| EAT | 0.85 | 0.90 |

ICC, intraclass correlation efficient.

**Supplementary Table S2.** Subgroup analysis of biomarkers according to LA size

| Variables | Enlarged LA* (n=51) | Without enlarged LA (n=32) | p value |
| --- | --- | --- | --- |
| Age, year | 72 (62, 76) | 61 (54, 68) | **< 0.001** |
| Male (%) | 26 (63) | 25 (78) | **0.013** |
| BMI, kg/m2 | 26.7±3.4 | 25.7±2.8 | 0.159 |
| Glucose, mmol/l | 6.3 (5.9, 7.4) | 6.2 (5.4, 7.4) | 0.194 |
| PsAF (%) | 32 (78) | 11 (26) | **0.012** |
| HTN (%) | 30 (73) | 22 (52) | 0.363 |
| CAD (%) | 13 (32) | 11 (26) | 0.385 |
| PVD (%) | 12 (29) | 10 (24) | 0.438 |
| DM (%) | 17 (41) | 14 (33) | 0.340 |
| Stroke (%) | 14 (34) | 6 (14) | 0.367 |
| Serum biomarkers | | | |
| hsCRP, pg/mL | 1.4 (0.6, 2.8) | 1.2 (0.7, 2.4) | 0.978 |
| BNP, mg/L | 204 (114.5, 289.0) | 60.5 (24.8, 145.0) | **< 0.001** |
| LDL, mmol/L | 2.3±0.8 | 2.3±0.8 | 0.943 |
| Echocardiogram | | | |
| RA area, cm^2^ | 21.6±4.1 | 17.7±4.2 | **< 0.001** |
| LVEF, % | 64.1±5.0 | 67.1±7.4 | **0.035** |
| EAT activity | 1.5 (1.3, 1.7) | 1.3 (1.2, 1.5) | **0.003** |
| LA activity | 2.1 (1.8, 2.6) | 1.9 (1.7, 2.2) | **0.027** |
| LAA activity | 2.0 (1.8, 2.6) | 1.7 (1.5, 1.9) | **< 0.001** |
| RA activity | 2.6 (2.0, 3.3) | 1.9 (1.7, 2.1) | **< 0.001** |
| Inflammatory markers | | | |
| IL-6, pg/ml | 5.9 (3.9, 8.2) | 5.9 (4.4, 8.2) | 0.581 |
| IL-8, pg/ml | 15.2 (5.8, 24.1) | 14.2 (5.7, 25.1) | 0.379 |
| IL-10, pg/ml | 10.1±6.6 | 13.9±7.3 | **0.014** |
| IL-18, pg/ml | 42.8 (34.2, 77.6) | 61.6 (38.4, 92.6) | 0.105 |
| TNF-α, pg/ml | 4.5 (1.9, 12.1) | 6.3 (3.8, 12.4) | 0.185 |
| Hsp27, ng/ml | 139.7±69.2 | 139.9±77.4 | 0.989 |
| Hsp60, ng/ml | 46.2±17.7 | 58.4±19.8 | **0.004** |
| Hsp70, ng/ml | 21.2±11.0 | 25.9±12.4 | 0.073 |
| Fibrotic markers | | | |
| TGF-β1, ng/ml | 8.4±2.5 | 9.4±2.8 | 0.07 |
| PDGF-BB, ng/ml | 3.3 (2.8, 3.4) | 3.4 (2.6, 5.3) | 0.199 |
| sST2, ng/ml | 12.1±5.8 | 14.2±6.8 | 0.139 |
| Gal-3, ng/ml | 6.9 (5.7, 9.3) | 7.2 (5.2, 9.6) | 0.975 |
| MMP-2, ng/ml | 247.8±98.7 | 243.9±109.5 | 0.865 |
| MMP-9, ng/ml | 1097.9±610.7 | 1081.0±597.3 | 0.533 |
| MPO, ng/ml | 1248.5±369.2 | 1109.5±341.7 | 0.079 |

BMI, body mass index; LAVI, left atrium volume index; RA, right atrium; SUVmax, maximum standardized uptake value; EAT, epicardial adipose tissue

*LA enlargement was defined as LAVI > 28 ml/m^2(1)^

1. Abhayaratna WP, Seward JB, Appleton CP, Douglas PS, Oh JK, Tajik AJ, et al. Left atrial size: physiologic determinants and clinical applications. J Am Coll Cardiol 2006; 47: 2357-63.

**Supplementary Table S3.** Subgroup analysis of biomarkers according to RA size

| Variables | Enlarged RA* (n=64) | Without enlarged RA (n=19) | p value |
| --- | --- | --- | --- |
| Age, year | 69 (58, 75) | 60 (56, 68) | 0.078 |
| Male (%) | 43 (67) | 8 (42) | **0.049** |
| BMI, kg/m2 | 26.5±3.4 | 25.3±1.8 | **0.048** |
| Glucose, mmol/l | 6.5 (5.8, 7.5) | 5.9 (5.4, 5.8) | **0.040** |
| PsAF (%) | 42 (66) | 1 (5) | **< 0.001** |
| HTN (%) | 44 (69) | 8 (42) | **0.035** |
| CAD (%) | 17 (27) | 7 (37) | 0.385 |
| PVD (%) | 18 (28) | 4 (21) | 0.540 |
| DM (%) | 25 (39) | 6 (32) | 0.554 |
| Stroke (%) | 17 (27) | 3 (16) | 0.510 |
| Serum biomarkers | | | |
| hsCRP, pg/mL | 1.4 (0.6, 2.7) | 1.0 (0.7, 2.4) | 0.329 |
| BNP, mg/L | 150.0 (86.3, 278.5) | 28.0 (15.0, 68.0) | **< 0.001** |
| LDL, mmol/L | 2.2 (1.7, 2.7) | 2.5 (2.3, 3.1) | **0.046** |
| Echocardiogram | | | |
| LAVI, cm^3^ | 29.7 (26.1, 33.1) | 16.0 (15.2, 17.6) | **< 0.001** |
| LVEF, % | 64.4±6.5 | 69.8±4.8 | **0.001** |
| EAT activity | 1.4 (1.2, 1.5) | 1.3 (1.2, 1.6) | 0.658 |
| LA activity | 2.1 (1.7, 2.4) | 1.9 (1.8, 2.1) | 0.094 |
| LAA activity | 1.9 (1.7, 2.3) | 1.6 (1.5, 1.9) | **0.007** |
| RA activity | 2.2 (1.9, 3.0) | 1.8 (1.6, 2.2) | **0.004** |
| Inflammatory markers | | | |
| IL-6, pg/ml | 5.9 (3.8, 8.2) | 5.9 (5.3, 8.5) | 0.392 |
| IL-8, pg/ml | 14.2 (6.7, 24.4) | 16.3 (9.7, 28.5) | 0.324 |
| IL-10, pg/ml | 11.5±7.6 | 13.9±5.6 | 0.213 |
| IL-18, pg/ml | 42.8 (34.9, 72.6) | 80.6 (52.7, 110.8) | **0.008** |
| TNF-α, pg/ml | 5.3 (2.7, 11.9) | 6.5 (3.3, 12.5) | 0.461 |
| Hsp27, ng/ml | 145.9±71.9 | 119.4±75.1 | 0.166 |
| Hsp60, ng/ml | 53.2±20.4 | 49.8±17.3 | 0.515 |
| Hsp70, ng/ml | 22.8±12.2 | 25.9±11.1 | 0.318 |
| Fibrotic markers | | | |
| TGF-β1, ng/ml | 8.8±2.7 | 9.3±2.6 | 0.460 |
| PDGF-BB, ng/ml | 3.3 (2.6, 4.4) | 4.3 (3.2, 6.3) | 0.076 |
| sST2, ng/ml | 12.7±6.2 | 14.8±6.7 | 0.206 |
| Gal-3, ng/ml | 7.0 (5.2, 9.6) | 7.0 (5.8, 9.1) | 0.558 |
| MMP-2, ng/ml | 243.9±99.7 | 251.9±118.9 | 0.770 |
| MMP-9, ng/ml | 1179.4±616.7 | 1007.1±544.1 | 0.276 |
| MPO, ng/ml | 1125.8 (865.7, 1446.2) | 1232.3 (986.6, 1344.7) | 0.324 |

BMI, body mass index; LAVI, left atrium volume index; RA, right atrium; SUVmax, maximum standardized uptake value; EAT, epicardial adipose tissue

*RA enlargement was defined as RA area > 15 cm^2^ for women and > 16 cm^2^ for men ^(1)^

1. Grünig E, Henn P, D'Andrea A, Claussen M, Ehlken N, Maier F, et al. Reference values for and determinants of right atrial area in healthy adults by 2-dimensional echocardiography. Circ Cardiovasc Imaging 2013; 6: 117-124.

**Supplementary Table S4.** Subgroup analysis of biomarkers according to age

| Variables | > 65 years (n=51) | <= 65 years (n=32) | p value |
| --- | --- | --- | --- |
| BMI, kg/m2 | 25.5 (24.7, 27.3) | 25.8 (23.7, 28.7) | 0.891 |
| Glucose, mmol/l | 6.3 (5.7, 7.7) | 6.3 (5.4, 7.1) | 0.256 |
| PsAF (%) | 25 (49) | 18 (56) | 0.521 |
| HTN (%) | 31 (61) | 21 (66) | 0.657 |
| CAD (%) | 17 (33) | 7 (22) | 0.262 |
| PVD (%) | 14 (27) | 8 (25) | 0.686 |
| DM (%) | 19 (37) | 12 (38) | 0.982 |
| Stroke (%) | 15 (29) | 5 (16) | 0.153 |
| Serum biomarkers | | | |
| hsCRP, pg/mL | 1.4 (0.7, 2.7) | 1.2 (0.6, 2.7) | 0.982 |
| BNP, mg/L | 172.0 (65.0, 279.0) | 95.3 (23.0, 209.0) | **0.045** |
| LDL, mmol/L | 2.1±0.7 | 2.5±0.8 | **0.016** |
| Echocardiogram | | | |
| LAVI, cm^3^ | 30.9 (26.3, 34.4) | 23.9 (17.1, 29.8) | **0.001** |
| RA area, cm^2^ | 20.3±4.3 | 19.0±4.8 | 0.206 |
| LVEF, % | 64.4±6.2 | 66.9±6.7 | 0.071 |
| EAT activity | 1.4 (1.2, 1.6) | 1.4 (1.2, 1.6) | 0.978 |
| LA activity | 2.1 (1.9, 2.3) | 1.9 (1.7, 2.3) | 0.303 |
| LAA activity | 1.9 (1.7, 2.2) | 1.8 (1.6, 2.2) | 0.421 |
| RA activity | 2.2 (1.8, 2.9) | 2.0 (1.7, 2.8) | 0.349 |
| Inflammatory markers | | | |
| IL-6, pg/ml | 5.9±2.5 | 6.5±3.4 | 0.413 |
| IL-8, pg/ml | 16.3 (6.7, 24.0) | 13.0 (87.,25,0) | 0.956 |
| IL-10, pg/ml | 12.9±7.9 | 11.1±6.3 | 0.236 |
| IL-18, pg/ml | 51.9 (34.9, 84.6) | 55.7 (35.7, 87.1) | 0.566 |
| TNF-α, pg/ml | 4.5 (2.3, 12.2) | 6.4 (3.3, 12.3) | 0.171 |
| Hsp27, ng/ml | 138.7±68.3 | 140.9±78.7 | 0.890 |
| Hsp60, ng/ml | 46.9 (36.9, 63.3) | 56.2 (39.5, 66.4) | 0.162 |
| Hsp70, ng/ml | 22.9 (13.6, 33.6) | 19.5 (13.5, 32.1) | 0.834 |
| Fibrotic markers | | | |
| TGF-β1, ng/ml | 8.8 (7.4, 10.4) | 8.6 (6.6, 12.1) | 0.953 |
| PDGF-BB, ng/ml | 3.3 (2.7, 4.4) | 3.3 (2.3, 5.2) | 0.709 |
| sST2, ng/ml | 13.2±6.2 | 13.1±6.6 | 0.914 |
| Gal-3, ng/ml | 6.9 (5.0, 9.1) | 7.1 (5.6, 9.6) | 0.809 |
| MMP-2, ng/ml | 262.6±101.9 | 227.7±103.7 | 0.126 |
| MMP-9, ng/ml | 1130.4±608.1 | 1150.3±603.4 | 0.881 |
| MPO, ng/ml | 1134.8 (951.7, 1344.7) | 1124.3 (865.7, 1537.7) | 0,848 |

BMI, body mass index; LAVI, left atrium volume index; RA, right atrium; SUVmax, maximum standardized uptake value; EAT, epicardial adipose tissue

**Supplementary Table S5.** Subgroup analysis of biomarkers according to gender

| Variables | Male (n=51) | Female (n=32) | p value |
| --- | --- | --- | --- |
| Age, year | 63 (53, 73) | 68 (61, 76) | 0.074 |
| BMI, kg/m2 | 26.8±3.2 | 25.3±2.7 | **0.032** |
| Glucose, mmol/l | 6.3 (5.5, 7.5) | 6.2 (5.7, 7.3) | 0.892 |
| PsAF (%) | 29 (57) | 14 (44) | 0.245 |
| HTN (%) | 32 (63) | 20 (63) | 0.982 |
| CAD (%) | 16 (31) | 8 (25) | 0.533 |
| PVD (%) | 14 (27) | 8 (25) | 0.805 |
| DM (%) | 20 (39) | 11 (34) | 0.657 |
| Stroke (%) | 13 (25) | 7 (22) | 0.708 |
| Serum biomarkers | | | |
| hsCRP, pg/mL | 1.3 (0.7, 2.2) | 1.3 (0.7, 3.1) | 0.660 |
| BNP, mg/L | 124.0 (59.0, 224.0) | 139.0 (36.1, 338.8) | 0.397 |
| LDL, mmol/L | 2.2±0.7 | 2.5±0.9 | 0.074 |
| Echocardiogram | | | |
| LAVI, cm^3^ | 28.4 (20.2, 32.7) | 27.7 (18.1, 33.9) | 0.863 |
| RA area, cm^2^ | 20.4±4.1 | 18.5±5.1 | 0.087 |
| LVEF, % | 64.5±5.9 | 67.4±7.2 | 0.050 |
| EAT activity | 1.4 (1.3, 1.5) | 1.3 (1.1, 1.6) | 0.172 |
| LA activity | 2.0 (1.7, 2.4) | 1.9 (1.9, 2.2) | 0.735 |
| LAA activity | 1.9 (1.7, 2.2) | 1.9 (1.6, 2.2) | 0.560 |
| RA activity | 2.0 (1.8, 2.8) | 2.1 (1.7, 3.0) | 0.966 |
| Inflammatory markers | | | |
| IL-6, pg/ml | 6.1±3.1 | 6.3±2.7 | 0.781 |
| IL-8, pg/ml | 16.0 (8.6, 25.4) | 12.6 (6.5, 21.9) | 0.290 |
| IL-10, pg/ml | 10.7±6.3 | 14.2±8.0 | 0.032 |
| IL-18, pg/ml | 42.8 (33.4, 76.6) | 61.6 (37.4, 91.6) | 0.163 |
| TNF-α, pg/ml | 5.9 (2.9, 12.6) | 5.2 (2.8, 12.4) | 0.594 |
| Hsp27, ng/ml | 147.8 (97.6, 199.5) | 96.6 (54.8, 143.3) | **0.002** |
| Hsp60, ng/ml | 51.9±19.3 | 53.0±20.6 | 0.816 |
| Hsp70, ng/ml | 21.1 (11.6, 32.2) | 24.3 (17.5, 33.9) | 0.192 |
| Fibrotic markers | | | |
| TGF-β1, ng/ml | 8.7±2.5 | 9.3±2.9 | 0.304 |
| PDGF-BB, ng/ml | 3.6 (3.1, 5.3) | 3.1 (2.3, 4.6) | **0.030** |
| sST2, ng/ml | 13.7±6.8 | 12.2±5.6 | 0.290 |
| Gal-3, ng/ml | 7.2 (5.8, 9.1) | 6.9 (4.9, 9.6) | 0.885 |
| MMP-2, ng/ml | 249.7±107.7 | 239.5±58.2 | 0.663 |
| MMP-9, ng/ml | 1157.5±560.9 | 1112.1±671.2 | 0.741 |
| MPO, ng/ml | 1149.5±369.7 | 1123.8±358.8 | 0.364 |

BMI, body mass index; LAVI, left atrium volume index; RA, right atrium; SUVmax, maximum standardized uptake value; EAT, epicardial adipose tissue

**Supplement Table S6.** Comparison between AF patients achieved successful AF termination by RFCA and those without

| Variables | Yes (n = 25) | No（n = 9） | *P* value |
| --- | --- | --- | --- |
| Age, years | 63±11 | 72±8 | **0.028** |
| Male (%) | 16 (67) | 7 (78) | 0.847 |
| BMI, kg/m^2^ | 26.1 (24.9, 28.7) | 25.3 (24.6, 27.1) | 0.376 |
| Blood glucose, mmol/L | 124.2±19.8 | 151.2±50.4 | 0.202 |
| Hypertension (%) | 19 (79) | 6 (67) | 0.772 |
| Coronary artery disease (%) | 5 (21) | 1 (11) | 0.502 |
| Peripheral vascular disease (%) | 3 (13) | 1 (11) | 0.913 |
| Diabetes mellitus (%) | 11 (46) | 7 (78) | 0.092 |
| Stroke (%) | 4 (17) | 3 (33) | 0.572 |
| Echocardiogram | | | |
| LAVI, ml/m^2^ | 30.5±5.7 | 31.0±4.2 | 0.808 |
| RA area, cm^2^ | 22.4±3.8 | 22.3±3.4 | 0.920 |
| LVEF, % | 63.5±6.4 | 60.1±6.1 | 0.167 |
| Voltage index | 2 (1, 3) | 4 (3, 5) | **0.001** |
| SUVmax-EAT | 1.2 (1.4, 1.6) | 1.5 (1.4, 1.6) | 0.102 |
| LA activity | 1.1 (1.0, 1.4) | 1.0 (0.9, 1.0) | **0.017** |
| LAA activity | 1.1 (1.0, 1.3) | 1.0 (0.9, 1.1) | 0.298 |
| RA activity | 1.4 (1.2, 1.7) | 1.1 (0.9, 1.4) | **0.041** |
| Serum biomarkers | | | |
| hsCRP, pg/mL | 1.4 (0.5, 3.0) | 1.2 (0.7, 2.1) | 0.376 |
| LDL, mmol/L | 2.3±0.8 | 1.9±0.9 | 0.309 |
| BNP, mg/L | 164.0 (122.0, 345.0) | 150.0 (88.5, 212.0) | 0.969 |
| IL-6, pg/ml | 7.5±3.1 | 5.7±2.8 | 0.134 |
| IL-8, pg/ml | 12.2 (5.2, 22.1) | 20.9 (10.8, 20.1) | 0.246 |
| IL-10, pg/ml | 12.2 (4.2, 18.2) | 11.2 (5.3, 11.9) | 0.465 |
| IL-18, pg/ml | 54.6±27.4 | 60.0±36.2 | 0.647 |
| TNF-α, pg/ml | 7.9±5.4 | 6.2±4.7 | 0.392 |
| Hsp27, ng/ml | 152.5±85.9 | 152.0±61.1 | 0.988 |
| Hsp60, ng/ml | 54.3±22.2 | 49.5±20.3 | 0.574 |
| Hsp70, ng/ml | 21.7±12.0 | 29.7±11.7 | 0.092 |
| TGF-β1, ng/ml | 8.6±2.8 | 8.4±2.5 | 0.841 |
| PDGF-BB, ng/ml | 3.3 (3.1, 4.9) | 4.3 (3.0, 5.2) | 0.397 |
| sST2, ng/ml | 12.9±6.0 | 15.2±8.2 | 0.393 |
| Gal-3, ng/ml | 7.9±2.8 | 7.3±3.4 | 0.546 |
| MMP-2, ng/ml | 251.2±106.2 | 280.4±112.1 | 0.492 |
| MMP-9, ng/ml | 1219.5±459.5 | 1102.1±405.8 | 0.661 |
| MPO, ng/ml | 1180.6±383.1 | 1072.9±301.7 | 0.543 |

BMI, body mass index; LAVI, left atrium volume index; RA, right atrium; LVEF, left ventricular ejection fraction; SUVmax, maximum standardized uptake value; EAT, epicardial adipose tissue

**Supplement Table S7.** Cox regression analysis of predictor of AF recurrence

| Variables | HR (95%CI) | *P* value |
| --- | --- | --- |
| PsAF (%) | 0.520 (0.195, 1.386) | 0.191 |
| Covariables: PsAF | | |
| Age, year | 1.038 (0.985, 1.093) | 0.160 |
| Male (%) | 1.304 (0.449, 3.791) | 0.626 |
| BMI, kg/m^2^ | 1.022 (0.873, 1.197) | 0.783 |
| Glucose, mmol/l | 0.856 (0.583, 1.258) | 0.429 |
| Hypertension (%) | 0.746 (0.276, 2.017) | 0.564 |
| Coronary artery disease (%) | 1.488 (0.542, 4.085) | 0.440 |
| Peripheral vascular disease (%) | 3.582 (1.274, 10.070) | **0.016** |
| Diabetes mellitus (%) | 0.885 (0.310, 2.522) | 0.810 |
| Stroke (%) | 0.909 (0.260, 3.174) | 0.881 |
| Echocardiography | | |
| LAVI, ml/m^2^ | 1.057 (0.992, 1.127) | 0.087 |
| RA area, cm^2^ | 1.069 (0.936, 1.220) | 0.326 |
| LVEF, % | 0.978 (0.902, 1.060) | 0.588 |
| SUVmax-EAT | 2.453 (0.673, 8.945) | 0.174 |
| LA activity | 1.052 (0.243, 4.500) | 0.946 |
| LAA activity | 0.621 (0.104, 3.701) | 0.601 |
| RA activity | 0.963 (0.360, 2.581) | 0.941 |
| Serum biomarkers | | |
| hsCRP, pg/mL | 0.572 (0.321, 1.019) | 0.058 |
| BNP, mg/L | 0.999 (0.996, 1.002) | 0.594 |
| LDL, mmol/L | 0.934 (0.504, 1.729) | 0.808 |
| IL-6, pg/ml | 0.834 (0.681, 1.021) | 0.079 |
| IL-8, pg/ml | 0.971 (0.933, 1.011) | 0.158 |
| IL-10, pg/ml | 0.940 (0.874, 1.012) | 0.099 |
| IL-18, pg/ml | 1.007 (0.995, 1.019) | 0.229 |
| TNF-α, pg/ml | 0.925 (0.833, 1.027) | 0.144 |
| Hsp27, ng/ml | 0.999 (0.993, 1.006) | 0.798 |
| Hsp60, ng/ml | 0.998 (0.975, 1.023) | 0.889 |
| Hsp70, ng/ml | 1.010 (0.974, 1.048) | 0.586 |
| TGF-β1, ng/ml | 0.998 (0.134, 1.194) | 0.983 |
| PDGF-BB, ng/ml | 1.098 (0.798, 1.511) | 0.566 |
| sST2, ng/ml | 0.916 (0.839, 1.001) | 0.053 |
| Gal-3, ng/ml | 1.024 (0.871, 1.206) | 0.772 |
| MMP-2, ng/ml | 1.001 (0.997, 1.006) | 0.530 |
| MMP-9, ng/ml | 1.000 (0.999, 1.001) | 0.715 |
| MPO, ng/ml | 1.000 (0.999, 1.002) | 0.892 |

BMI, body mass index; LAVI, left atrium volume index; RA, right atrium; SUVmax, maximum standardized uptake value; EAT, epicardial adipose tissue

**Supplement Table S8.** Comparison between AF with- and without early recurrence

| Variables | Recurrence (n=14) | Without recurrence (n=56) | *P* value |
| --- | --- | --- | --- |
| PsAF | 11 (78) | 23 (41) | **0.012** |
| Age, year | 65 (52, 70) | 67 (56, 74) | 0.304 |
| Male (%) | 9 (64) | 36 (64) | 1.0 |
| BMI, kg/m2 | 26.8±3.0 | 26.0±3.2 | 0.421 |
| Glucose, mmol/l | 6.7 (5.7, 7.4) | 6.3 (5.7, 7.3) | 0.597 |
| Hypertension (%) | 8 (57) | 18 (32) | 0.083 |
| Coronary artery disease (%) | 2 (14) | 18 (32) | 0.321 |
| Peripheral vascular disease (%) | 2 (14) | 15 (27) | 0.531 |
| Diabetes mellitus (%) | 6 (43) | 19 (34) | 0.533 |
| Stroke (%) | 2 (14) | 12 (21) | 0.823 |
| Echocardiogram | | | |
| LAVI, ml/m^2^ | 28.8±5.3 | 26.0±7.9 | 0.226 |
| RA area, cm^2^ | 21.4±3.6 | 18.9±4.7 | 0.073 |
| LVEF, % | 64±6 | 65±7 | 0.051 |
| EAT activity | 1.5 (1.2, 1.6) | 1.3 (1.2, 1.5) | 0.239 |
| LA activity | 2.2 (1.7, 2.8) | 1.9 (1.8, 2.3) | 0.309 |
| LAA activity | 1.9 (1.8, 2.6) | 1.9 (1.6, 2.1) | 0.218 |
| RA activity | 2.6 (1.9, 3.3) | 2.0 (1.7, 2.6) | 0.058 |
| Serum biomarkers | | | |
| hsCRP, pg/mL | 0.8 (0.6, 2.6) | 1.1 (0.6, 2.2) | 0.843 |
| BNP, mg/L | 127.0 (50.5, 280.2) | 118.0 (33.0, 260.7) | 0.592 |
| LDL, mmol/L | 2.5±1.1 | 2.2±0.7 | 0.401 |
| IL-6, pg/ml | 5.3 (4.2, 8.3) | 6.2 (4.3, 8.2) | 0.959 |
| IL-8, pg/ml | 14.1 (5.9, 24.3) | 15.7 (9.7, 25.3) | 0.378 |
| IL-10, pg/ml | 11.7±9.8 | 12.8±6.7 | 0.719 |
| IL-18, pg/ml | 57.2 (38.9, 116.9) | 51.7 (35.5, 82.0) | 0.343 |
| TNF-α, pg/ml | 4.1 (2.1, 8.8) | 5.7 (2.7, 12.3) | 0.336 |
| Hsp27, ng/ml | 146.7±82.2 | 133.6±68.9 | 0.545 |
| Hsp60, ng/ml | 52.1±23.0 | 53.1±19.8 | 0.877 |
| Hsp70, ng/ml | 27.9 (21.5, 34.7) | 20.6 (13.3, 33.3) | 0.199 |
| TGF-β1, ng/ml | 9.1±2.2 | 9.1±2.9 | 0.977 |
| PDGF-BB, ng/ml | 4.2 (2.9, 5.3) | 3.3 (2.5, 4.6) | 0.523 |
| sST2, ng/ml | 13.6±7.2 | 13.1±6.5 | 0.819 |
| Gal-3, ng/ml | 5.9 (5.6, 9.2) | 7.2 (5.3, 9.5) | 0.686 |
| MMP-2, ng/ml | 235.7±105.5 | 254.3±103.5 | 0.552 |
| MMP-9, ng/ml | 1374.2 (549.4, 1810.8) | 977.1 (662.4, 1523.4) | 0.450 |
| MPO, ng/ml | 881.4 (806.3, 1236.9) | 1125.8 (899.3, 1421.9) | 0.121 |

BMI, body mass index; LAVI, left atrium volume index; RA, right atrium; SUVmax, maximum standardized uptake value; EAT, epicardial adipose tissue

**Supplemental Table S9.** Comparisons between patients with- and without early recurrence

| Variables | With recurrence  (n = 8) | Without recurrence (n =18) | *P* value |
| --- | --- | --- | --- |
| Post LA | 0.7±0.1 | 0.8±0.1 | 0.012 |
| Post LAA | 1.1±0.3 | 1.1±0.1 | 0.692 |
| Post RA | 1.1±0.2 | 1.1±0.2 | 0.514 |
| Post EAT | 1.3±0.2 | 1.2±0.2 | 0.570 |
| Change of LA | 0.4 (0.3, 1.3) | 0.3 (0.1, 0.4) | 0.338 |
| Change of LAA | 0.2 (0.1, 0.3) | 0.0 (-0.1, 0.3) | 0.216 |
| Change of RA | 0.5 (0.3, 0.8) | 0.4 (0.1, 0.7) | 0.196 |
| Change of EAT | 0.3 (-0.1, 0.3) | 0.3 (-0.1, 0.4) | 0.765 |

LA, left atrium; LAA, left atrial appendage; RA, right atrium.

**Supplemental Table S10.** Comparisons between patients with- and without late recurrence

| Variables | With recurrence  (n = 5) | Without recurrence (n =21) | *P* value |
| --- | --- | --- | --- |
| Post LA | 0.7±0.1 | 0.8±0.1 | 0.313 |
| Post LAA | 1.2±0.3 | 1.1±0.2 | 0.188 |
| Post RA | 1.1±0.2 | 1.1±0.2 | 0.916 |
| Post EAT | 1.2±0.2 | 1.2±0.2 | 0.888 |
| Change of LA | 0.4 (0.3, 0.9) | 0.4 (0.3, 0.7) | 0.753 |
| Change of LAA | 0.1 (-0.2, 0.4) | 0.1 (0.0, 0.3) | 0.224 |
| Change of RA | 0.6 (0.3, 1.2) | 0.4 (0.2, 0.6) | 0.659 |
| Change of EAT | 0.3 (-0.15, 1.1) | 0.0 (-0.1, 0.2) | 0.950 |

LA, left atrium; LAA, left atrial appendage; RA, right atrium.
